# Effects of the COVID-19 pandemic on trauma-related emergency medical service in older people: a retrospective cohort study

**DOI:** 10.1186/s12873-023-00874-y

**Published:** 2023-08-26

**Authors:** Mohammadreza Sabbaghi, Kheizaran Miri, Mohammad Namazinia

**Affiliations:** 1 Department of Medical Emergency, School of Nursing and Midwifery, Torbat Heydariyeh University of Medical Sciences, Torbat Heydariyeh, Iran; 2 Department of Nursing, School of Nursing and Midwifery, Torbat Heydariyeh University of Medical Sciences, Torbat Heydariyeh, Iran

**Keywords:** COVID-19, EMS, Trauma, Older people

## Abstract

**Introduction:**

The ever-increasing human life expectancy has currently resulted in a noticeable rise in the world’s older population. Addressing the healthcare needs of the older people has become a significant concern for many countries. Moreover, the older people are particularly vulnerable to traumatic events. This study aimed to examine the impact of the COVID-19 pandemic on prehospital care provided by Emergency Medical Services (EMS) for trauma-related cases among the older people in Iran.

**Methods:**

This retrospective cohort study involved analyzing the medical records of 1,111 older people aged above 60 who experienced traumatic injuries and received pre-hospital emergency services from March 2018 to March 2022. In order to collect the data, the checklist made by the researcher was used and data analysis in SPSS16 was done using the Chi-square test and Fisher’s exact test.

**Results:**

The age group of 60–74 received the highest number of services both before and after the COVID-19 pandemic. The older men experienced more traumatic events compared to women throughout the study period. The majority of the traumatic events occurred between 8 a.m. and 12 p.m. both before and after the COVID-19 pandemic.

**Conclusions:**

The high prevalence rate of geriatric traumas can be primarily attributed to their physical problems and no control over movements caused by old age, as well as unsafe living conditions. To address these issues, it is suggested that facilities be provided to assist with mobility problems. Moreover, constructing suitable pedestrian bridges and regularly checking neighborhoods and surroundings to identify potential risk factors should be prioritized. Once these risk factors are identified, efforts can be made to adjust and eliminate them, thereby minimizing traumatic events and enhancing a safe and friendly environment for the older people.

## Introduction

The global increase in human life expectancy has contributed to a significant rise in the older population worldwide [1]. In the United States, for instance, over 49 million individuals, accounting for approximately 15% of the population, are currently aged 65 or above, a number projected to double by 2050 [2]. Similarly, the aging population in Iran is expected to reach 11.3% by 2025, and 31% by 2050 [3].

Old age is typically characterized by weakness or fatigue, accelerated cognitive decline, psychological challenges, low energy, as well as chronic and debilitating illnesses. As a result, the well-being of the older people has become a pressing public health concern for many countries [1, 2]. Evidence indicates that older people are at high risk of traumas [3, 4], to the extent that trauma-related mortality rates tend to be higher in this population compared to younger patients. Consequently, there is an urgent need for increased medical attention in trauma centers and emergency rooms to cater to the specific needs of the aging population. It is worth noting that the financial burden associated with caring for older people who have experienced trauma exceeds that of other diseases [5–7].

Iran, as a developing country in the Eastern Mediterranean region, is facing high rates of trauma-induced mortality and complications in older people [6, 8]. These rates surpass those reported in other countries [9, 10]. Trauma is the fourth leading cause of death in people aged 55–64, and the ninth leading cause of mortality in those aged 65 and above in Iran. Notably, 25% of all trauma-related deaths in the country occur in this older age group. Thus, it is projected that by 2050, approximately 40% of trauma patients will be older individuals [5].

Geriatric trauma is currently classified based on causal mechanisms such as traffic accidents, falls, or violence, as well as the affected body region and the characteristics of the causative agents, including penetrating, non-penetrating injuries, or barotrauma [11]. The mechanisms of trauma are observed to vary globally [12, 13]. In Iran, for instance, traffic accidents have been identified as the most common cause of trauma in the general population, followed by other mechanisms [14, 15]. Common causes of trauma in older people include vision loss, hearing disorders, physical disabilities, use of medications, altered sensory processing, and chronic and debilitating diseases like diabetes or heart disease [16–20].

Moreover, global epidemics, including the ongoing COVID-19 pandemic, can have a significant effect on the type and mechanism of trauma in the older people [21–23]. Although over two years have passed since the start of the COVID-19 pandemic, many aspects of the disease remain unknown to healthcare systems. Despite quarantine policies and reduced transportation and traffic during this pandemic, managing traumatic injuries in older people has posed challenges in terms of delivery of prehospital care and emergency medical services (EMS) [24, 25].

Some studies conducted before the COVID-19 pandemic in the United Kingdom, Canada, and the United States have reported varying prevalence rates of falls in the aging population, approximately 30%, 20%, and 12%, respectively [26]. In addition, a study conducted during the COVID-19 pandemic in Turkey identified traffic accidents as the most common cause of trauma among the older people [27].

It is worth noting that the characteristics and mechanisms of trauma observed in studies focusing on younger populations may differ significantly from those relevant to older individuals, who often require more frequent prehospital care EMS missions. Moreover, to the best of the authors’ knowledge, no studies have investigated the impact of the COVID-19 pandemic on trauma in the older people in Iran. In light of these circumstances, this study aimed to examine the impact of COVID-19 on trauma-related prehospital care EMS among older people in Iran.

## Methods

### Study setting and design

We conducted a retrospective cohort study that included all EMS calls made in the city of Torbat-e Heydarieh, Razavi Khorasan, Iran, with a population of about one million people. The study period was from March 2018 to March 2022.

### Populations

The statistical population consisted of 1111 older people who had interacted with the prehospital care EMS centers in Torbat-e Heydarieh from March 2018 to March 2022. It is important to note that the sampling method employed was census sampling, meaning that the entire population of interest was included in the study.

### Measurement instruments

The data collection tool was a researcher-made checklist that focused on epidemiological indicators specific to the older people. The checklist consisted of two parts. The first part gathered information on demographic characteristics such as gender, age, and place of living. The second part included details on prehospital care EMS mission time, main complaints, mission outcomes, and types of contact with the prehospital care EMS centers. This tool had been already administered in some surveys to investigate the causes of prehospital care EMS calls [28, 29]. The validity of the checklist was assessed using the content validity index (CVI = 0.9) to ensure its accuracy and relevance to the study objectives. To establish the CVI, the checklist was initially reviewed by 10 faculty members specialized in nursing and prehospital care EMS, who evaluated its content and provided suggestions for improvements. The tool reliability in this study was confirmed by completing its time indicators with responses from 30 respondents. To measure internal consistency, Cronbach’s alpha coefficient was calculated, resulting in a coefficient of 0.81.

### Sampling

Upon receiving the code of ethics, the researchers accessed the raw data from the ASAYAR software, which is a smart system used for managing and controlling the process of prehospital care EMS delivery in Iran. This software program has been operational in the prehospital care EMS centers in Torbat-e Heydarieh since March 2018.

### Statistical analysis

The data were then analyzed using the SPSS16. Descriptive statistics (frequency distribution, mean, and standard deviation [SD]) were used to describe and categorize the data. Additionally, the Chi-square test and Fisher’s exact test were employed to compare the variables before and during the COVID-19 pandemic. The normality of quantitative data was assessed using the Kolmogorov-Smirnov statistic. Noteworthy, a confidence interval (CI) of 95% and a significance level of 0.05 were considered in all tests.

## Results

**Fig. 1:**
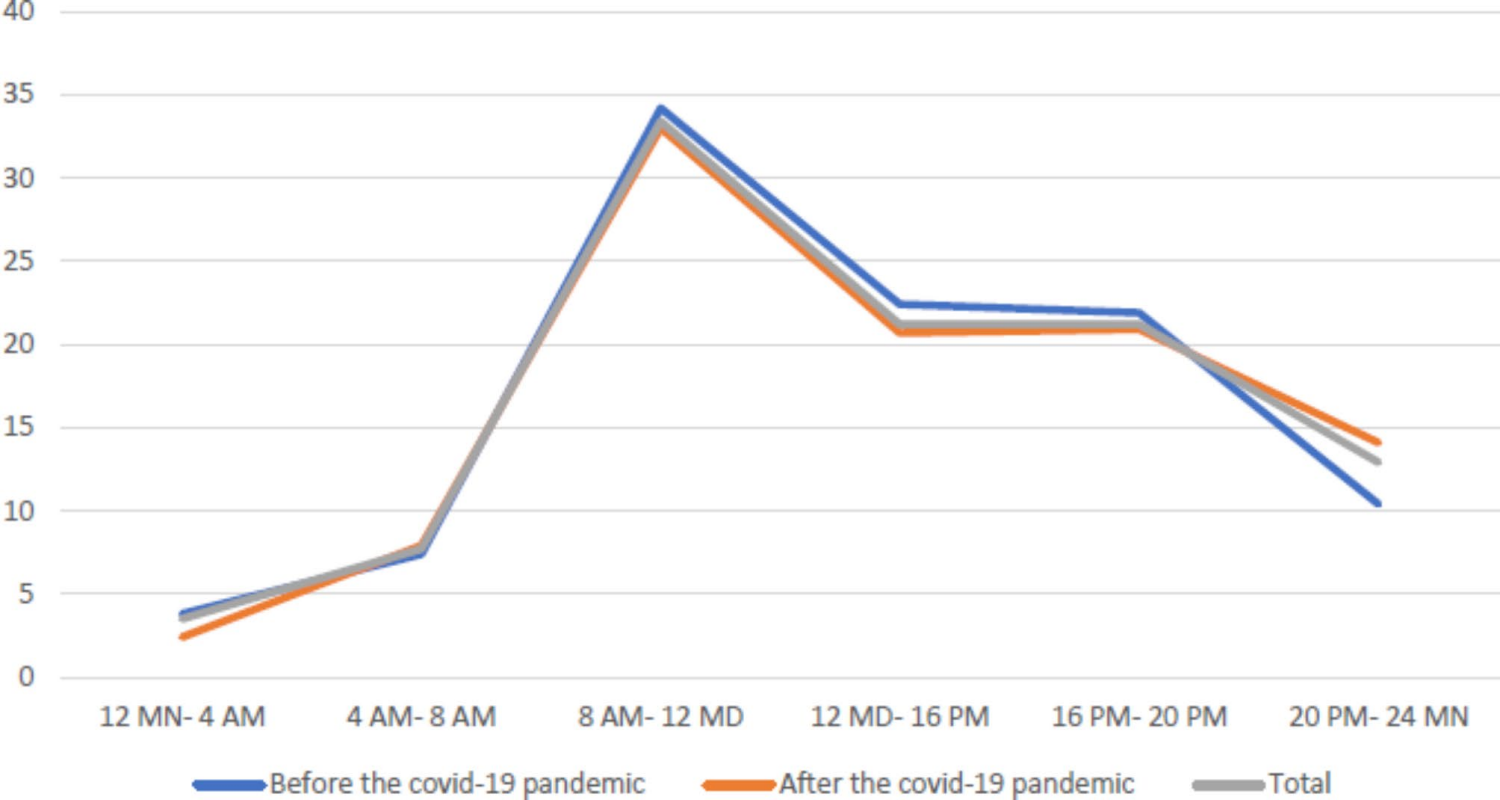
The percentage of trauma recorded in the elderly in the period before and after the covid-19 pandemic according to time intervals in 24 h

The study results revealed that 1111 prehospital care EMS missions in Torbat-e Heydarieh were related to older people. The highest number of missions occurred during the COVID-19 pandemic (n = 745 cases). The age group of 60–74 received the highest number of services both before and after the pandemic. Throughout the study period, older men experienced more traumas compared to women. The majority of traumatic events, both before and after the COVID-19 pandemic, happened between 8 a.m. and 12 p.m. (Fig. 1). Before the pandemic, the older people had a higher incidence of traumas in spring, whereas after the pandemic, the incidence increased in summer. Most of the reported traumas affecting the older people occurred in urban areas. Moreover, nearly all cases with traumas, both before and after the COVID-19 pandemic, preferred visiting the prehospital care EMS centers in person rather than calling them (p < 0.001) (Table 1).

**Table 1:** Characteristics of the elderly assisted by pre-hospital emergency in the period before and during the covid-19 pandemic

Variable			Before the Covid-19 pandemic n = 366	during the covid-19 pandemic n = 745	Total n = 1111
Age, years (± SD)		60–74	233 (63.7)	460 (61.7)	P = 0.260
		75–89	121 (33.1)	244 (32.8)	
		90<	12 (3.3)	41 (5.5)	
Gender n (%)		Male	240 (65.6)	499 (67.0)	P = 0.637
		Female	126 (34.4)	246 (33.0)	
Time of mission n (%)		12MN- 4 AM	14 (3.8)	25 (2.4)	P = 0.637
		4AM-8AM	27 (7.4)	59 (7.9)	
		8AM-12MD	125 (34.2)	246 (33.0)	
		12MD-16PM	82 (22.4)	154 (20.7)	
		16PM-20PM	80 (21.9)	156 (20.9)	
		20PM-24MN	38 (10.4)	105 (14.1)	
Season n (%)		Spring	111 (30.3)	151 (20.3)	P < 0.001
		Summer	108 (29.5)	219 (29.4)	
		Autumn	88 (24.0)	180 (24.2)	
		Winter	59 (16.1)	195 (26.2)	
The mission area n (%)		City	364 (99.5)	728 (92.7)	P = 0.046
		Road	2 (0.5)	17 (2.3)	
Type of trauma, n(%)	Road Traffic Accident		185 (50.5)	359 (48.2)	P < 0.001
	Fall		130 (35.5)	283 (38.0)	
	Animal attack		36 (9.8)	10 (1.3)	
	strife		12 (3.3)	85 (11.4)	
	burn		3 (0.8)	8 (1.1)	
Mission result n(%)	Transfer		343 (93.7)	634 (85.1)	P < 0.001
	Primary measures and non-transmission		23 (6.3)	111 (14.9)	
Type of contact with pre-hospital emergency n(%)	By phone		319 (87.2)	699 (93.8)	P < 0.001
	In person		47 (12.8)	46 (6.2)	

Before the COVID-19 pandemic, out of the total prehospital care EMS missions, 343 cases (93.7%) resulted in the injured individuals being transported to medical facilities. However, 23 cases (6.3%) were not transferred to medical facilities for various reasons such as receiving initial emergency care or not giving consent for transfer. After the COVID-19 pandemic, 634 cases (85.1%) resulted in the injured individuals being sent to medical facilities, while 111 cases (14.9%) were not transferred for reasons similar to those before the pandemic (Table 1) (p < 0.001).

**Fig. 2:**
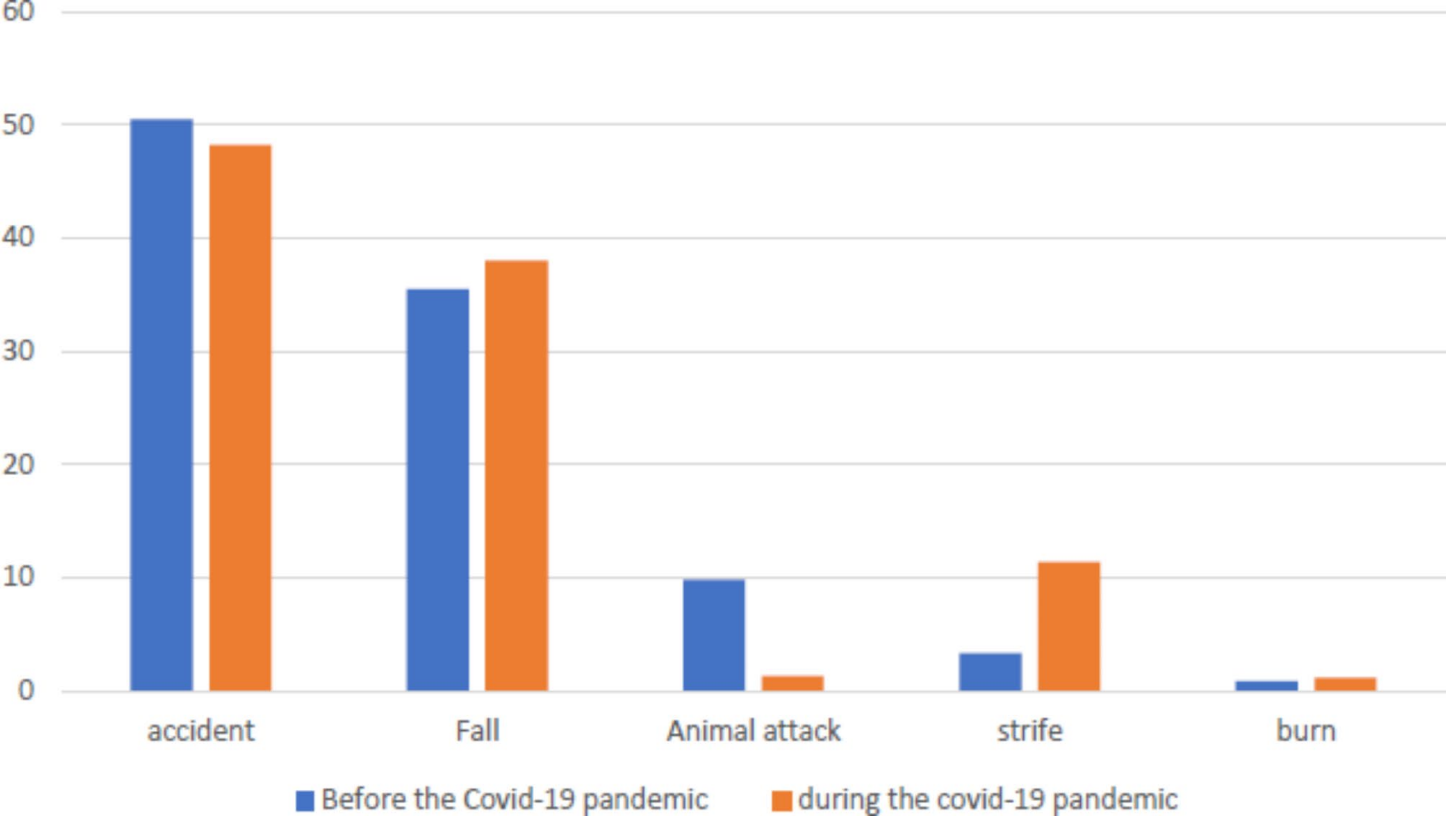
The type of trauma in the elderly according to the time period before and after the covid-19 pandemic

Traffic accidents were the most common mechanism of trauma among the older people both before and after the COVID-19 pandemic. However, in non-traffic accidents, falls, animal bites, fights, and burns were the main causes of trauma. It is worth noting that after the COVID-19 pandemic, there was a noticeable decrease in the occurrence of animal bites compared to before the pandemic (Table 1) (Fig. 2) (p < 0.001).

## Discussion

The present study aimed to investigate the impact of the COVID-19 pandemic on trauma-related prehospital care EMS among the older people in eastern Iran. The study findings demonstrated a significant difference in the type of trauma, season, mission area, mission outcome, and type of contact with the prehospital care EMS centers before and during the pandemic in this region.

Consistent with previous surveys conducted in Iran and Turkey, traffic accidents were identified as the most common cause of trauma in the aging population before and for the period of the COVID-19 pandemic [28, 29]. Accordingly, traffic accidents have been reported as the leading cause of trauma and the second cause of mortality in Iran [30, 31]. The country has experienced a higher death rate related to traffic accidents compared to other nations, approximately 30 cases per 100,000 population, which has been increasing in recent years [27, 32]. Considering the older population in Iran, there is a higher likelihood of involvement of pedestrians, passengers, and the older motorists in traffic accidents. Age itself is considered a major risk factor that may increase the number and severity of traumas [33, 34]. Other causes of trauma among the older people include vision loss, hearing disorders, physical disabilities, use of medications, altered sensory processing, and chronic and debilitating diseases like diabetes or heart disease [4, 16].

The second leading cause of geriatric traumas both before and during the COVID-19 pandemic was falls, which aligns with findings reported in previous surveys in Iran [3, 27, 29], South Korea, Pakistan [4, 35], United Kingdom, Canada, and the United States. Various studies have reported prevalence rates of falls in the older people, ranging from approximately 12–30% [26]. Old age is characterized by accelerated cognitive decline, balance problems, loss of muscle mass and strength, and reduced coordination of lower limbs, which collectively increase the risk of falls among older people [36, 37]. Additionally, previous history of falls, daily routines, concomitant illnesses, and use of medications are major factors that can contribute to geriatric falls [38].

In addition to traffic accidents and falls, the third cause of trauma in the older population before the COVID-19 pandemic was animal bites. However, during the pandemic, fights emerged as the third cause of trauma. The animals responsible for causing traumas in the older people were dogs, snakes, cats, scorpions, and bees, with dog bites being the most frequent. It is worth noting that there has been a sharp rise in the number of traumas due to animal bites in Europe prior to the COVID-19 pandemic [39]. In addition, between 2005 and 2009, approximately 1.6 million cases of traumas caused by dog bites were treated in emergency rooms in the United Stated [40]. During the COVID-19 pandemic, the older population became more vulnerable due to their physical conditions, susceptibility to the virus, and the implementation of quarantine measures, making them the primary targets of such incidents [41]. Nevertheless, the risk of traumas after animal bites in public places has decreased.

The fourth cause of trauma in the older people during the COVID-19 pandemic was fights. Some studies have highlighted the issue of violence and abuse against the older people (including physical violence, psychological violence, sexual abuse, neglect, and financial burdens) [42, 43]. The risk factors associated with this increase in \ violence during the pandemic included long-term home quarantine, fear of contagion, stress, misinformation on social networks, limited access to services, despair, fatigue, insufficient income, lack of information, unemployment, financial losses, and limited social support [44]. These factors paved the ground for violence and abuse against the older population, which aligns with the findings in the present study.

In this study, traumas were more common in the older men before and during the COVID-19 pandemic, which is consistent with the results of other domestic and international studies [29, 45, 46]. This higher prevalence of traumas in older men can be attributed to their engagement in high-risk behaviors, including work injuries, assaults, sports-related incidents, and accidents, which corroborates the findings of the current study. In addition, the study observed that probability of traumas among the older people was higher from 8 a.m. to 12 p.m. This finding was consistent both before and during the COVID-19 pandemic and can be attributed to the fact that this time period coincided with rush hours in the city, thus increasing the risk of accidents [47].

In the present study, traumas were more prevalent in the spring and summer seasons before and during the COVID-19 pandemic. This could be attributed to the increased trend of trips and the presence of older people in parks, recreational centers, and public spaces during these seasons [48]. These results support the results of numerous studies conducted nationally and internationally [9, 49, 50]. Furthermore, the likelihood of traumas among the older people was higher in urban areas than the rural ones. A study conducted in Iran highlighted that one of the leading causes of trauma in urban areas was the older pedestrians [51]. Similar research in third-world countries has also emphasized the importance of pedestrian safety, particularly for the aging population [51–54]. This finding aligns with the results of the present study.

In this study, the majority of the older people had contacted prehospital care EMS center via phone before and during the COVID-19 pandemic. Throughout the study period, most of the older people were transferred to medical facilities for further care and treatment. Notably, the prehospital care EMS centers in Iran play a crucial role in the healthcare system, providing services to people of different nationalities, ethnic groups, and cultures in normal and emergency situations. These services can be accessed simply by calling 115 [55]. The prehospital care EMS services can be provided to the older people in various settings, including their own homes, nursing homes, business centers, recreational centers, and other places.

This study had several limitations that should be taken into consideration. First, the researchers were unable to investigate the cases where the older people were referred to medical facilities by their family members. Second, this study did not include the older people who were injured at home but did not seek medical care. Third, the study did not report the exact number and severity of traumas in the aging population before and during the COVID-19 pandemic.

## Conclusion

The high prevalence rate of geriatric traumas can be attributed to their physical problems and no control over movements that often accompany old age. Unsafe living conditions also contribute to these injuries. The present study revealed that traffic accidents and falls were the most common causal mechanisms of trauma in the older population before and during the COVID-19 pandemic. It is suggested to provide facilities to deal with mobility problems in the older population. This can include measures such as constructing suitable pedestrian bridges and checking neighborhoods and surroundings to identify risk factors. Efforts should be made to adjust and eliminate these risk factors, with the aim of minimizing traumatic events and providing a safe and friendly environment for older people.

## Data Availability

The datasets generated in the current study are available from the corresponding author upon reasonable request.

## Abbreviations

EMS: Emergency medical services
